# Coordinated terrestrial locomotion in California grunion ( *Leuresthes tenuis* )

**DOI:** 10.17912/micropub.biology.001431

**Published:** 2025-04-11

**Authors:** Clinton Moran, Alice Gibb, Kathryn Dickson

**Affiliations:** 1 Biology, Citadel, Charleston, South Carolina, United States; 2 Biology, Northern Arizona University, Flagstaff, Arizona, United States; 3 U.S. National Science Foundation, Alexandria, Virginia, United States

## Abstract

Terrestrial locomotor behaviors in fishes allow fishes to escape biotic and abiotic pressures in aquatic habitats. Emerging to spawn once a year, California grunion beach themselves during Spring high tides. We described two coordinated behaviors that adult grunion consistently perform during short, but regular, bouts of movement on land: the tail-flip jump and “sidestepping” behavior. While tail-flip jumping covered more ground per event, repeated sidestepping allowed grunion to cover comparable ground per unit time. This is the first published kinematic description of grunion locomotor behaviors in terrestrial habitats based on video recordings.

**Figure 1. Behavioral description of grunion moving on land:**
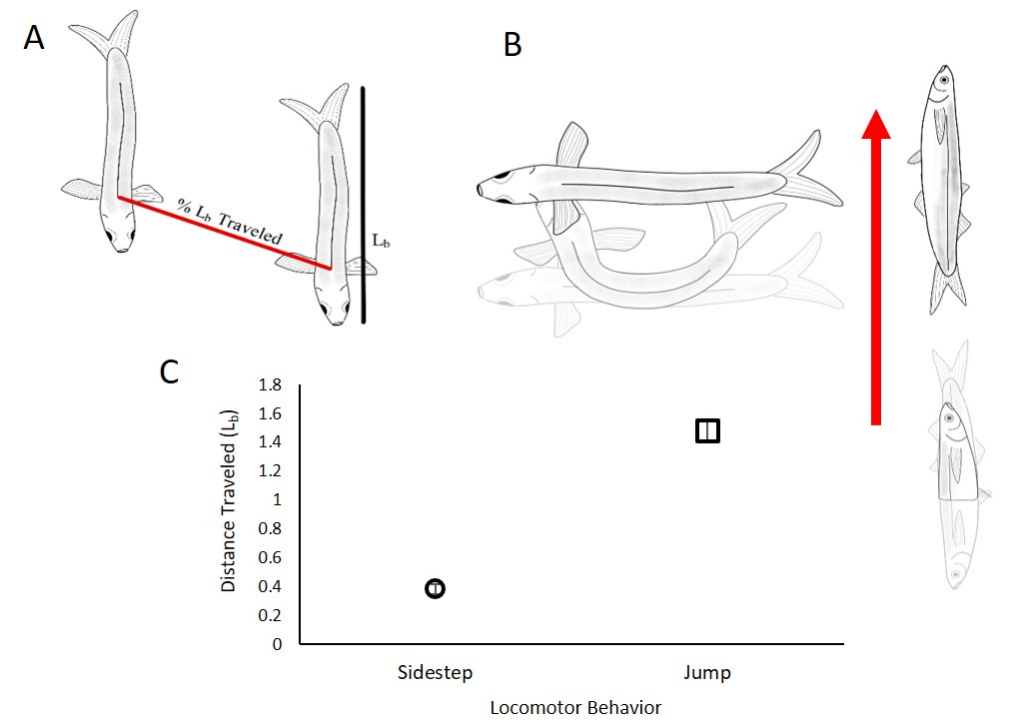
Methods and data representing terrestrial locomotor differences for grunion on land. A: Distance traveled was standardized to body length (L _b_ ) of the fish measured in pixels from high-speed videos. Distance traveled, using the approximate center of mass, was measured from the start of the behavior until a single bout was completed. B: sidestepping (left; n=50) and tail-flip jumping (right; n=30) behaviors illustrated with the red arrow indicating the direction of travel. C: Average (±s.e.m.) distance covered (L _b_ ) for a single behavior was greater in the tail-flip jump compared to sidestepping.

## Description

More than 60 fish species experience an amphibious period during their life cycle. Biotic (e.g., prey capture, predation, reproduction, competition) or abiotic (ex. temperature, salinity, O _2_ availability) stressors may force fish to leave the water (Sayer and Davenport 1991). During their time on land, amphibious fishes are exposed to physiological and locomotor demands not experienced in aquatic environments (Gordon et al., 1969). Fishes that regularly spend time on land, however, have developed behavioral adaptations to enhance their abilities to move in a terrestrial environment (e.g., Gibb et al., 2013).

Across amphibious fish lineages, the degree of specialization increases with the amount of time spent in terrestrial habitats (Axlid et al., 2023). Fishes that very rarely experience emersion produce uncoordinated behaviors (e.g. “fish out of water” thrashing) when stranded on land. Those that experience occasional emersion as part of their life histories have been shown to produce a coordinated “tail-flip jump,” characterized by the head and anterior body curling up over the body, followed by straightening of the axial body which pushes the tail and caudal peduncle against the substrate, launching the fish into the air (Gibb et al., 2011). Finally, some terrestrial-specialist species exhibit a “prone jump,” which is a directed maneuver from the prone (lying on ventral aspect) position, where the posterior body and tail are brought forward, placed on the ground near the head, and used to propel the fish into an anteriorly-directed ballistic jump (Swanson and Gibb, 2004; Gibb et al., 2013).

California grunion ( *Leuresthes tenuis* ) (henceforth: grunion) is a beach-spawning species (Martin and Swiderski, 2001). Individuals advance with the incoming tide to spawn and lay eggs in the intertidal zone during spring high tides (Walker, 1952). On land, female grunion bury the posterior portion of their bodies in the sand and deposit eggs, which are fertilized by surrounding males. Following reproduction, males and females return to the ocean by traversing over sand and, sometimes, over other grunion. While there is extensive literature on the ecology and reproduction of grunion, to our knowledge there is no published study quantifying their terrestrial locomotor behaviors.

Our goal was to classify terrestrial locomotor behaviors performed by grunion during their intertidal breeding activities. We considered two possible findings. (1) Because grunion spend the majority of their lives in the ocean, they might only be able to produce uncoordinated movements on land. In this scenario, the distances that grunion have to move are relatively small, so even “thrashing” type movements, in combination with wave action, could allow spawning and a return to the ocean. (2) Alternately, because this is a critical time for grunion in their life history, coordinated behaviors may have evolved to facilitate movements over land.

We imaged grunion moving on land during a night spawn on 4/1/2014 at Cabrillo Beach (CA, USA). Using clips from these recordings, behaviors of individual grunion were observed, categorized, and quantified. We measured the displacement of the approximate center of mass for each observed behavior; displacement of each fish was standardized to its body length ( Fig. 1A ), producing movement in units of length (L _b_ ).

At times, grunion exhibited uncoordinated behaviors that were not repeated with consistency. However, despite their nearly exclusively aquatic life history, we observed grunion to consistently produce two forms of coordinated movements on land: (1) a tail-flip jump, and (2) a distinct behavior we term “sidestepping”. These locomotor modes involved stereotyped (repeated behaviors, featuring distinct characteristics) movements, produced displacement of the fish, and were observed repeatedly in most individuals.

As has been seen in other species, the tail-flip jump is characterized by the animal lying on its side, lifting its head and anterior above the tail, and pushing off using the tail and caudal peduncle to launch into ballistic flight (Gibb et al., 2011). Sidestepping is characterized by the animal laying on its ventral surface, curling the head and tail into a ‘C' shape, then using the head and tail as anchor points to move the rest of the axial body laterally through the ‘C' shape, then forming another ‘C' on the contralateral side of the body ( Fig. 1B ). We measured body displacement for 50 sidestepping events (from 20 individuals) and 30 tail-flip jumps (from 30 individuals).

The tail-flip jump resulted in an average (L _b_ ±s.e.m) displacement of 1.48 ± 0.08. A single bout of sidestepping behavior resulted in an average (L _b_ ±s.e.m) displacement of 0.39 ± 0.03. Tail-flip jumping results in greater body displacement than does a single bout of sidestepping (Student's t-test, *P<0.001* ) ( Fig. 1C ). Grunion often performed the sidestepping behavior consecutively three to four times before stopping. Conversely, following a tail flip jump, the fish would either lay motionless or reposition for another behavior (coordinated―sidestep or jump or uncoordinated―flopping). Effectively this would allow fish performing repeated sidestepping to move a comparable distance to the tail-flip jump per unit time. Anecdotally, the fish we measured did not have a precise direction they would move while performing these behaviors. However, if the beach were split in half (towards ocean and away from ocean) most of the behaviors we observed would have been directed towards the ocean. We believe these fish swim up the beach with incoming waves, deposit gametes in the sand, and return to the water with these behaviors.

Gibb et al. (2013) quantified variation in ability to produce an effective tail-flip jump across a variety of teleost taxa. Fishes considered proficient tail-flip jumpers move between 4 and 7 body lengths in a single ballistic event (Gibb et al. 2013). The body displacement from jumps by grunion did not come close to this mark; grunion only travel ~1.5 L _b_ per jump. Despite being a relatively “poor” performer, it is important to consider the ecological implications of jumping on land for grunion. Spawning events occur with thousands of fish beaching themselves, depositing gametes in the sand and returning to the ocean. During a spawning “swarm,” the tail-flip jump allows a fish to propel itself vertically, over other individuals, to reach a potential mate or to return to the water. By traveling through the air, the grunion do not have to navigate around other fish when moving up or down the beach.

The relatively poor displacement from the tail-flip jump may be due to differences in body shape between “good jumpers” and grunion. Minicozzi et al. (2020) found that fish species that produced large displacement during a tail-flip jump had length to depth relationships (body fineness ratio, following Webb, 1975) of 4-7, with good jumpers confined to a fineness ratio between 3-5. The only good jumper that has a fineness ratio >5 is *Kryptolebias marmoratus,* a species that has posteriorly placed dorsal and anal fins that assist in the tail-flip jump (Axlid et al., 2013). From individual video images, we calculated the average fineness ratio for grunion in this study to be 7.3. To our knowledge, this is the highest fineness ratio recorded for a fish performing a tail-flip jump and might reflect the upper morphological limit for this behavior. The grunion's slender body shape and lack of morphological specializations for jumping suggests that individuals of this species are better suited for hydrodynamic swimming performance than for terrestrial locomotor movements.

During spawning, grunion traverse the beach, move over one another other, and insert themselves into the sand using a variety of movements, many of which appear to have no predictability or stereotypy. However, in addition to the tail-flip jump, grunion also utilize a sidestepping motion to move on land. This movement is distinct from anguilliform movements described for the terrestrial locomotion of elongate fishes such as eels ( *Anguilla rostrata* ) (Gillis, 1998; Gillis, 2000; Gillis and Blob, 2001), ropefish ( *Erpetoichthys calabaricus* ) (Pace and Gibb, 2011) and snakehead ( *Channa argus* ) (Bressman et al., 2019). Instead, it is more similar to the sidewinding behavior demonstrated by snakes. In snakes, horizontal broadside movement is facilitated by anchor points along the axial body (Jayne, 2020), whereas anchor points for grunion sidestepping are the head and tail. Recently, Kuznetsov (2022) reported a similar behavior in the elongate Asian swamp eel ( *Monopterus albus)* , with the overall movement of the center of mass in the anterior direction. In contrast, for sidestepping grunion, the overall direction of the center of mass is in the lateral direction ( Fig. 1B ).

Another feature of sidestepping behavior in grunion is that it often occurs in sequential cycles. From the videos, individuals that jumped stayed motionless for several seconds before attempting to move again. Thus, while sidestepping in grunion amounts to less distance traveled than a tail-flip jump, it may add up to a greater overall distance traveled because multiple sidestepping movements can be completed repeatedly in close succession. However, the effectiveness of sidestepping may be limited on the often-crowded beaches, as grunion must contact the surface to execute the behavior and the presence of other individuals may inhibit their overland progress.

This study demonstrates that individuals of a fish species, California grunion ( *Leuresthes tenuis* ), which spend most of their lives in the water can still produce effective overland movements during short periods of emersion. Grunion, an elongate fish species that possesses no obvious specializations for terrestrial movement, is able to move across the wave-swept upper intertidal to perform behaviors that are key to mating success and individual fitness. However, grunion may be less efficient in moving on land than other species studied thus far, requiring more cycles of movement, and possibly greater energetic effort, to move a significant distance across the sand.

## Methods

Beached grunion were recorded at night using a Panasonic Lumix DMC-FZ200 12.1 MP Digital Camera with a frame rate of 120 frames s ^-1^ while being illuminated by overhead floodlights. Grunion were filmed in naturally occurring wave-swept habitats with variable densities of other spawning fish. Two behaviors were consistently observed and appeared to be coordinated. To characterize kinematics of behaviors, we measured distance traveled following one movement cycle. Using ImageJ (Abràmoff et al., 2004) 50 sidesteps and 30 jumps were recorded from different individuals. Distances traveled were recorded in body lengths (L _b_ ) using the approximate center of mass (COM, estimated as the most anterior point of the dorsal fin). A Student's t-test was used to compare distances traveled between each behavior. An alpha of 0.05 was used for all statistical analyses.

## Data Availability

Description: Clip of tail flip jump behavior in grunion . Resource Type: Audiovisual. DOI: https://doi.org/10.22002/fxez3-n9322 Description: The novel sidewinding behavior observed in grunion . Resource Type: Audiovisual. DOI: https://doi.org/10.22002/08a0r-6jh45
